# Contribution of mono and polysaccharides to heterotrophic N_2_ fixation at the eastern Mediterranean coastline

**DOI:** 10.1038/srep27858

**Published:** 2016-06-16

**Authors:** E. Rahav, M. J. Giannetto, E. Bar-Zeev

**Affiliations:** 1 Israel Oceanographic and Limnological Research, National Institute of Oceanography, Haifa, 8030, Israel; 2 Department of Chemical and Environmental Engineering, Yale University, New Haven, CT 06520, USA; 3 Zuckerberg Institute for Water Research, The Jacob Blaustein Institutes for Desert Research, Ben-Gurion University of the Negev, Sede Boqer Campus, 84990, Midreshet Ben-Gurion, Israel

## Abstract

N_2_ fixation should be a critical process in the nitrogen-poor surface water of the eastern Mediterranean Sea. Despite favorable conditions, diazotroph abundance and N_2_ fixation rates remains low for reasons yet explained. The main goal of this study was to investigate the limiting nutrients for diazotrophy in this oligotrophic environment. Hence, we conducted dedicated bottle-microcosms with eastern Mediterranean Sea water that were supplemented with mono and polysaccharides as well as inorganic nitrogen and phosphorous. Our results indicate that the diazotrophic community expressing *nifH* was primarily represented by heterotrophic Proteobacteria. N_2_ fixation and heterotrophic bacterial activity increased up-to tenfold following two days of dark incubations, once seawater was supplemented with organic carbon substrate in the form of glucose (monosaccharides) or gum-xanthan (polysaccharide surrogate). Furthermore, our results point that carbon-rich polysaccharides, such as transparent exopolymer particles, enhance heterotrophic N_2_ fixation, by forming microenvironments of intense metabolic activity, high carbon: nitrogen ratio, and possibly low O_2_ levels. The conclusions of this study indicate that diazotrophs in the eastern Mediterranean coast are primarily limited by organic carbon substrates, as possibly in many other marine regions.

N_2_ fixation is an important source of new bioavailable N in oligotrophic marine systems. Due to the high energetic requirements of N_2_ fixation^1^, most studies have focused on phototrophic, bloom-forming diazotrophs such as *Trichodesmium* spp.^2^ or unicellular cyanobacteria^3^ that can harvest light as an energy source for primary production. Yet, planktonic heterotrophic proteobacterial diazotrophs may also be important contributors to N_2_ fixation^4^,^5^ and are widely distributed throughout numerous marine environments^6^,^7^,^8^.

Due to the high metabolic requirements of diazotrophy^1^, N_2_-fixing organisms are often limited by different nutrients such as phosphorus^9^, iron^10^, or both^11^. Recent studies demonstrated that diazotrophs may also be limited by the availability of dissolved organic carbon in various marine environments including the southwest Pacific^12^,^13^, the eastern tropical south Pacific^14^ and in oxygen minimum zones^15^,^16^.

The surface water of the eastern Mediterranean Sea (EMS) is a sunlit, warm and impoverish environment, with low concentrations of dissolved nitrogen (N), phosphorus (P) and carbon (C) at the surface^17^,^18^. These conditions have assigned the EMS as an ideal environment for diazotrophy to occur. Concurrently, during previous decades, geochemical evidences from sapropels with extremely low δ^15^N^19^ and water samples with high N:P^20^ also suggested that N_2_ fixation should be widespread across the EMS. Indeed, diazotrophs are often detected in the EMS and were previously reported to be highly diverse, with representatives from all known clusters^21^,^22^,^23^. Yet, only low N_2_ fixation rates (0 to 0.4 nmol N L^−1^ d^−1^) have been measured throughout the EMS^18^,^23^,^24^,^25^,^26^, with only one sporadic *Trichodesmium* bloom event ever reported^27^. The discrepancy between the potentially favorable conditions for diazotrophy and the low N_2_ fixation rates with the absence of diazotrophs blooms in the EMS was previously explained by phosphorus and/or iron limitations^28^, with inconclusive and inconsistent responses or trends.

In this study, we focused on the role of monosaccharides (i.e. glucose) and polysaccharides (i.e. transparent exopolymer particles) as limiting factors for bacterial metabolism and diazotrophic activity in the oligotrophic EMS. To do so, we conducted nutrient enrichment bioassays using surface EMS water during summertime when oligotrophy is at its peak. Our results indicate that the active diazotrophs community comprised mostly heterotrophic Proteobacterial diazotrophs that were primarily limited by organic carbon substrates and not phosphorus as previously hypothesized. The conclusions of this research shed new light on the role of heterotrophic diazotrophy and their limiting factors in the EMS; one of the most oligotrophic marine environments in the world.

## Materials and Methods

### Water sample collection

Surface water were collected from the EMS (32° 49′34.86 N, 34° 57′23.11 E, Haifa, Israel) by pumping seawater from a shallow (~5 m) station during the summer months; September 2013, June and August 2014. Seawater was sampled for inorganic nutrients, chlorophyll *a,* heterotrophic bacterial abundance, primary and bacterial production, respiration, transparent exopolymer particles (TEP), β-glucosidase activity, N_2_ fixation and sequencing of the *nifH* gene (genomic DNA [gDNA] and complementary DNA [cDNA]).

### Nutrient addition experiment

Eight nutrient-enrichment microcosms (in biological triplicates) were carried out in 4.5L polycarbonate bottles using surface EMS water in September 2013. The incubation bottles were pre-washed with 10% hydrochloric acid and rinsed three times with Milli-Q water followed by three times with ambient seawater. Microcosms included (all concentrations are final): [1] no addition, [2] 0.1 μM K_2_HPO_4_ (P), [3] 1.6 μM NaNO_3_ (N), [4] 1 μM glucose (G), [5] NP, (6) GP, [7] GN and [8] GNP.

Microcosm bottles were incubated for 2 days in an outdoor pool with seawater flow-through to maintain ambient temperature (27–29 °C, Raveh *et al*.^18^) under natural light (representing full dial cycle) or under complete dark conditions. The darken bottles were also supplemented with a photosynthetic inhibitor 3-(3,4-dichlorophenyl)-1,1-dimethylurea (DCMU, 50 nM final concentration) to favor heterotrophic activity^29^.

### Polysaccharide-addition experiment

Gum-xanthan (GX) enriched microcosms (in biological triplicates) were conducted during August 2014 to elucidate the role of TEP as a labile carbon source for heterotrophic diazotrophy. Initially, surface EMS water was filtered through a 5-μm polycarbonate membrane filter (PALL Corp.) in order to retain most of the large aggregates. The pre-filtered water (4.5L) was either supplemented with gum-xanthan (300 μg L^−1^) or left unamended (controls) for two days under heterotrophic-favored conditions (i.e. dark and DCMU) as described above. GX was dissolved in deionized water (100 ml) and extensively homogenized (Thomas Scientific Model D1000) for 15 minutes as described by Rahav *et al*.^29^.

### Inorganic nutrients

Triplicate water samples were collected in 15-mL acid-washed plastic scintillation vials and were immediately frozen (−20 °C). Nutrient concentrations were measured using a segmented flow Technicon Auto-Analyzer II (AA-II) system^30^. The limits of detection (twice the standard deviation of the blank) were 0.08 μM for nitrate + nitrite and 0.008 μM for phosphate.

### Chlorophyll *a* extraction (chl.*a*)

Seawater samples (300 mL) were passed through a Whatman GF/F filter, extracted overnight in cold acetone (90%) under dark conditions and determined by the non-acidification method^31^ using a Turner Designs (Trilogy) fluorometer with 436 nm excitation and 680 nm emission filters.

### Primary production (PP)

Photosynthetic carbon fixation rates were estimated using the ^14^C incorporation method^32^. See supplementary information for further details.

### Bacterial production (BP)

Rates were estimated using the ^3^H-leucine (Amersham, specific activity: 160 Ci mmol^−1^) incorporation method^33^. A conversion factor of 1.5 kg C mol^−1^ per mole leucine incorporated was used, assuming an isotopic dilution of 2.0^34^. See supplementary information for further details.

### Heterotrophic bacterial abundance

Samples (1.8 mL) for bacterial abundance were collected, fixed with 50% glutaraldehyde (0.15% final conc. v/v., Sigma G7651), incubated in room temperature for 10 min, and frozen in liquid nitrogen until analyses. Prior to counting, samples were fast thawed at 37 °C and stained with 0.5 nM SYTO9 (Applied Biosystems) in the dark for 15 min^35^. Sub-samples (100 μL) were analyzed with an Attune acoustic focusing flow cytometer (Applied Biosystems) equipped with a syringe-based fluidic system at 408 and 488 nm wavelengths at a flow rate of 25 μl min^−1^. Beads (nominal size 0.93 μm) (Polysciences) were used as a size standard.

### Dissolved oxygen (DO) and bacterial respiration rate (BR)

Oxygen was measured with the Winkler titration method modified by Carpenter^36^. Water was immediately fixed with MnSO_4_ and KI + NaOH and sealed without headspace in 300 mL Winkler bottles (Wheaton). Once oxygen was residue, H_2_SO_4_ was added to the samples and titrated with Na_2_S_2_O_3_ using a Metrohm 785 DMP titrino auto-burette and double platinum electrode (end-point titration precision, ±1 μmol L^−1^).

Bacterial respiration (BR) rates were determined according to the following equation:


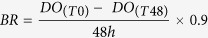


Where *DO*_(*T0*)_ is the initial dissolved oxygen concentration and *DO*_(*T48*)_ is the dissolved oxygen concentration after two days of dark incubation. We assumed that BR accounted for 90% of the entire community respiration as both chl.*a* and primary production decreased in the same proportion under dark (with DCMU) conditions.

### Bacterial carbon demand (BCD)

BCD was defined as the sum of carbon assimilation measured by bacterial production plus carbon oxidation through bacterial respiration. Oxygen respiration was converted into carbon consumption assuming a respiratory quotient (RQ) of 1^37^,^38^.

### Transparent exopolymer particles (TEP) concentration

Water samples (100 mL) were filtered through 0.4 μm polycarbonate filters (GE Water & Process Technologies) under low pressure (<150 mbar) and stained for 5 sec with a 0.02% Alcian Blue solution and 0.06% acetic acid (pH = 2.5). The excess dye was removed with a quick Milli-Q water rinse. TEP concentrations (μg gum-xanthan equivalents L^−1^) were measured according to Passow and Alldredge^39^. A conversion factor of 0.74 was used to convert from micrograms of gum-xanthan (GX) to the equivalent micrograms of carbon^40^.

### Beta-glucosidase activity (β-Glu)

The hydrolytic activity of β-glucosidase was determined by analysis of cleavage rates of a conjugated fluorogenic substrate, 4-methylumbelliferyl (MUF)-β-D-glucopyranoside (Sigma M3633) as described in Hoppe^41^. Briefly, substrate was added to a 1 mL water sample (final concentration of 50 μM) in triplicates and incubated in the dark at ambient temperature for one day. The increase in fluorescence was measured at 365 nm excitation, 455 nm emissions (GloMax^®^-Multi Detection System E9032) and calibrated against a MUF standard. A conversion factor of 72 nM MUF to μg carbon was applied as described in Bar-Zeev and Rahav^38^.

### N_2_ fixation analyses

Rates of N_2_ fixation were measured using the ^15^N_2_-enriched seawater method^42^. ^15^N_2_ enriched seawater was prepared by injecting 1:100 (v/v) ^15^N_2_ gas (99%, Cambridge Isotopes) into filtered (0.2 μm, PALL) and degassed (MiniModule G543) seawater collected at the study site. The enriched seawater stock was then vigorously shaken to completely dissolve the ^15^N_2_ gas, and aliquots (225 mL) were added to the experimental bottles (5% of total sample volume). After two days of incubation under either ambient light or complete dark conditions, the samples were filtered through pre-combusted (450 °C, 4.5 h) 25 mm Whatman GF/F and dried overnight in an oven at 60 °C. The samples were analyzed on a CE Instruments NC2500 elemental analyzer interfaced to a Thermo-Finningan Delta Plus XP isotope ratio mass spectrometer (IRMS). For isotope ratio mass spectrometry, a standard curve to determine N mass was generated for each sample run. Based on natural abundance, N mass on the filters, incubation times, and precision of the mass spectrometer, our calculated detection limit for ^15^N uptake was 0.02 nmol N L^−1^ d^−1^.

### Extraction and sequencing of the *nifH* gene

Samples (1L) were filtered through 0.2 μm Supor filters (PALL Corp.) and placed in a sterile DNase/Rnase Free Whirl-Pak bag. The samples were than snap frozen in liquid nitrogen and stored at −80 °C. DNA was extracted using the phenol-chloroform method according to Man-Aharonovich *et al*.^21^. RNA extraction was carried with a mirVana RNA isolation kit (Ambion). Genomic DNA (gDNA) contamination was removed using the DNase I digestion Turbo DNA-free kit (Ambion) and removal was verified by PCR (16S universal primers 519F-1492R) prior to reverse transcription. Complementary DNA (cDNA) synthesis was accomplished using a High Capacity cDNA Reverse Transcription Kit (Applied Biosystems) following manufacturer’s instructions.

Nitrogenase Fe protein transcripts (*nifH*) were amplified using a nested PCR strategy^43^. A paired-end sequencing of either DNA or cDNA was performed on an Illumina MiSeq platform at the Research and Testing Laboratories (Lubbock, TX, USA). See supplementary information for further details.

### Sequencing analysis

Merged Illumina reads were quality filtered and analyzed with the Quantitative Insights Into Microbial Ecology (QIIME) pipeline^44^. The remaining reads were binned into operational taxonomic units (OTUs), defined at 97% similarity, using the UCLUST algorithm^45^. Taxonomy was assigned with BLAST and a database of *nifH* sequences from Heller *et al*.^46^. Phylogenetic trees were generated with FastTree in QIIME^47^, and visualized with the Interactive Tree of Life (IToL) and Topiary Explorer v1.0 packages.

Many of the OTUs were unidentifiable, and therefore *nifH* sequences from representative phototrophs and heterotrophs were added to the trees as outgroups, using *Anabaena sphaerica* for rooting (Table S1). OTUs that clustered with either a phototrophic or a heterotrophic outgroup were considered phototrophs or heterotrophs, respectively. Further details on quality filtering criteria and sequencing data analyses are indicated in the supplementary information.

### Statistical analyses

Changes in bacterial production, primary production, bacterial carbon demand, β-glucosidase and N_2_ fixation rates in the different treatments were evaluated using a one-way analysis of variance (ANOVA), followed by a Fisher LSD multiple comparison *post hoc* test with a confidence of 95% (α = 0.05). A Pearson linear correlation was carried out between N_2_ fixation and bacterial cell specific activity and TEP (α = 0.05). All statistic tests were done using the XLSTAT software.

## Results and Discussion

### Physicochemical and biological characteristics of the study site

The coastal water was characterized by typical EMS summer conditions, with warm, saline and well-oxidized conditions (Table 1). Average dissolved inorganic nitrogen and phosphorus were in the lower end of oligotrophic environments^30^, resulting in basal chl.*a* concentrations (phytoplankton proxy) and low primary production rates (Table 1). Heterotrophic bacterial abundance and production rates remained unchanged in all sampling dates (Table 1) and were similar to previously reported summertime concentrations and rates in the EMS^18^,^48^.

**Figure 1:**
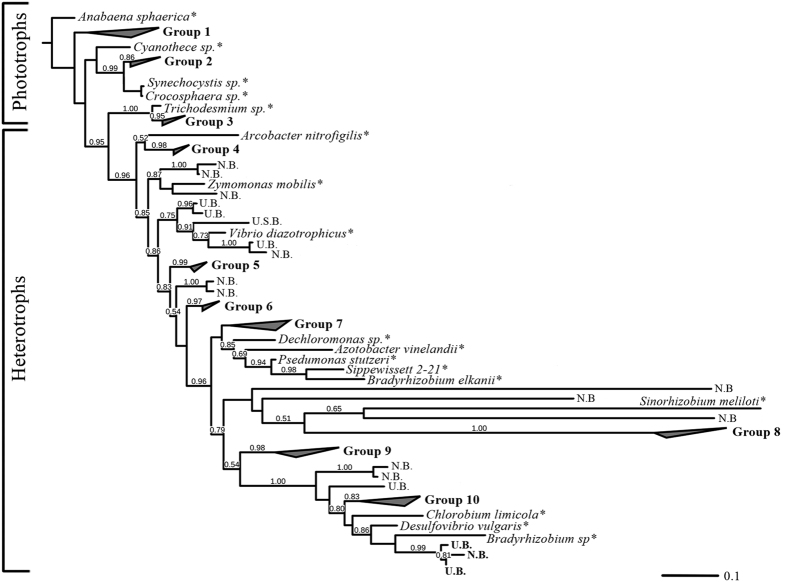
A phylogenetic tree of *nifH* gDNA OTUs obtained from the coastal EMS water. Bootstrap values exceeding 50% are indicated above the branches. Values are reported for neighbor-joining (NJ) analyses. The asterisk represents outgroups of common phototrophic and heterotrophic diazotrophs (more details in Table S1).

The diazotrophic community, based on *nifH* DNA analyses, was represented by both heterotrophic bacteria and autotrophic cyanobacteria (Fig. 1). The retrieved sequences converged into ten distinct groups ascribed to clusters I and III^49^. We acknowledge that since only 1-L of water was sampled, species with low abundances such as *Trichodesmium* may have been overlooked. Yet, based on previous studies in the EMS, bloom-forming N_2_ fixers such as *Trichodesmium* were rarely detected, even after the filtration of ~20-L of seawater^21^,^22^. Although the EMS is presumably an ideal environment for cyanobacterial diazotrophs (sunlit, warm and nitrogen-poor, Table 1), studies spanning over a decade have determined that most of the diazotrophs species cluster within the Proteobacterial clades, mainly composed of alpha, beta and gamma-proteobacteria^21^,^23^. Our results indicate that the diazotrophic diversity was highly compatible with long-term summer observations of the coastal and open EMS^21^,^22^,^23^.

Concurrent with the absence of bloom forming cyanobacterial diazotrophs, measured N_2_ fixation rates were overall low (Table 1) and similar to those measured in the open^24^,^25^,^26^,^50^ and coastal^18^ EMS water. Currently, the reasons for the absence of large bloom forming diazotrophs or the low N_2_ fixation rates in this nitrogen-poor system are unknown.

Phosphorus was the most studied limiting nutrient in the EMS due to the high N:P ratio in its deep water suggesting that diazotrophs are hindered by P availability. Therefore, only P, P + Fe or dust were examined to date as a diazotrophy-limiting nutrients in the surface of the EMS^26^,^28^. Yet, the addition of P yielded inconclusive responses with insignificant changes in N_2_ fixation rates across the Levantine Basin during July 2009^50^ and a 3-fold increase during June-July 2008^28^. Concurrently, dust additions were found to elevated N_2_ fixation via the release of P and Fe^28^ and/or by supplying airborne diazotrophs associate with the dust particles^51^.

A recent study across the Mediterranean Sea determined that N_2_ fixation measured under dark conditions equaled the rates retrieved under ambient light^50^. These results suggest that heterotrophic diazotrophs play a significant role in supplying new bioavailable nitrogen to the Mediterranean Sea^6^,^12^. Heterotrophic diazotrophs, unlike phototrophs (e.g. *Trichodesmium* and *Crocosphaera*), cannot harvest light through photosynthesis to maintain the energetic needs of the nitrogenase complex. Instead, these organisms scavenge and mineralize organic substrates. Assuming heterotrophic diazotrophs are important contributors to N_2_ fixation in the EMS^8^,^50^, we postulated that the prime factor that limits diazotrophy is organic carbon.

### Diazotrophic feedback to the addition of organic carbon

**Figure 2:**
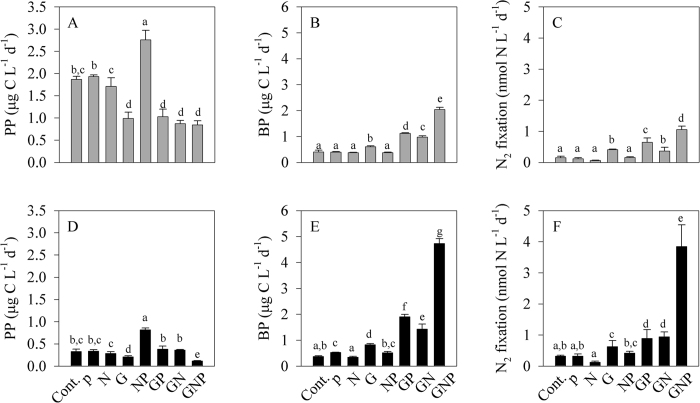
Physiological response of nutrient additions on N_2_ fixation. Bottle microcosms were incubated for 48 h with different nutrient additions, under ambient light (**A–C**) and dark +DCMU (**D–F**) conditions. Primary production, PP (**A,D**), bacterial production, BP (**B,E**) and N_2_ fixation rates (**C,F**) measured in response to various nutrient supplements. Control (Cont.); phosphorus (P); nitrogen (N); glucose (G); nitrogen + phosphorus (NP); glucose + phosphorus (GP); glucose + nitrogen (GN); glucose + nitrogen + phosphorus (GNP). The letters above the columns represent significant differences (one-way ANOVA and a Fisher LSD means comparison test, P < 0.05) for mean values between additions.

Our results indicate that N_2_ was primarily fixed by heterotrophic-bacterial diazotrophs under ambient light (Fig. 2A–C) and dark (Fig. 2D–F) conditions once organic carbon was not limiting. Incubating EMS water under ambient light conditions with the different nutrient additions did not affect phytoplankton concentrations (ranging from 0.27 to 0.43 μg chl.*a* L^−1^); apart than NP addition which triggered elevated phytoplankton biomass (1.60 μg chl.*a* L^−1^). These results are in agreement with previous studies from the EMS indicating that phytoplankton (not-necessarily diazotrophs) are co-limited by N and P^52^.

Similarly, no significant changes were found in heterotrophic bacterial abundance across the different microcosms (6 × 10^8^ to 8 × 10^8^ cells L^−1^, one-way ANOVA and a Fisher LSD means comparison test, P > 0.05). However, following glucose additions (G, GN, GP and GNP), primary production significantly decreased (Fig. 2A), while bacterial production drastically increased (Fig. 2B). These results suggest that glucose (as an organic carbon surrogate), rather than phosphorus^52^, is the prime limiting nutrient for heterotrophic bacterial activity at our study site (EMS). It is likely that once organic carbon is present and bacterial growth is prompt, both N and P become the limiting factors for bacterial activity. Further, we suggest that phytoplankton and heterotrophic bacteria are competing for the available nutrients as previously found in open sea EMS^48^,^52^.

At the end of the light incubations, N_2_ fixation rates were overall low (<0.5 nmol N L^−1^ d^−1^) and similar to values across the EMS^24^,^50^. However, when G was added (G, GN, GP, GNP), N_2_ fixation rates were enhanced by 2 to 7 fold compared with the control microcosms (Fig. 2C). The compatibility between the increased bacterial biomass and activity with the elevated N_2_ fixation rates indicate that diazotrophic heterotrophs are the prime N_2_-fixing organisms in our system once organic carbon is available. We surmise that the overall low N_2_ fixation rates, even under ambient light conditions that hypothetically should favor cyanobacterial diazotrophs, result from a competition with phytoplankton for any available N or P that are essential for bacterial metabolism.

In the experiment where microcosms were incubated under dark conditions, phytoplankton concentrations were low regardless of the supplements combinations and ranged between 0.10 to 0.23 μg chl.*a* L^−1^. Correspondingly, primary production rates were significantly lower than under the light condition, with negligible changes between the different microcosms, apart than NP (Fig. 2D). Similarly to the microcosm incubations conducted under ambient light, heterotrophic bacterial abundance remained unchanged, regardless of the different nutrient additions (6 × 10^8^ to 10 × 10^8^ cells L^−1^, one-way ANOVA and a Fisher LSD means comparison test, P > 0.05). However, bacterial production rates increased once glucose was added, and peaked (4.7 ± 0.2 μg C L^−1^ d^−1^) in the microcosms containing the GNP addition (Fig. 2E). N_2_ fixation rates, similarly to the bacterial production, were significantly higher following glucose additions and drastically increased (12 fold) with GNP supplements (Fig. 2F). Since phototrophic activity was suppressed by the long—dark incubations, it is highly likely that only heterotrophs and facultative microorganisms could utilize the available nutrients and proliferate. Hence, the high N_2_ fixation rates measured in the dark microcosms- especially following glucose addition (up to 3.84 nmol N L^−1^ d^−1^), were mostly attributed to heterotrophic diazotrophs.

**Figure 3:**
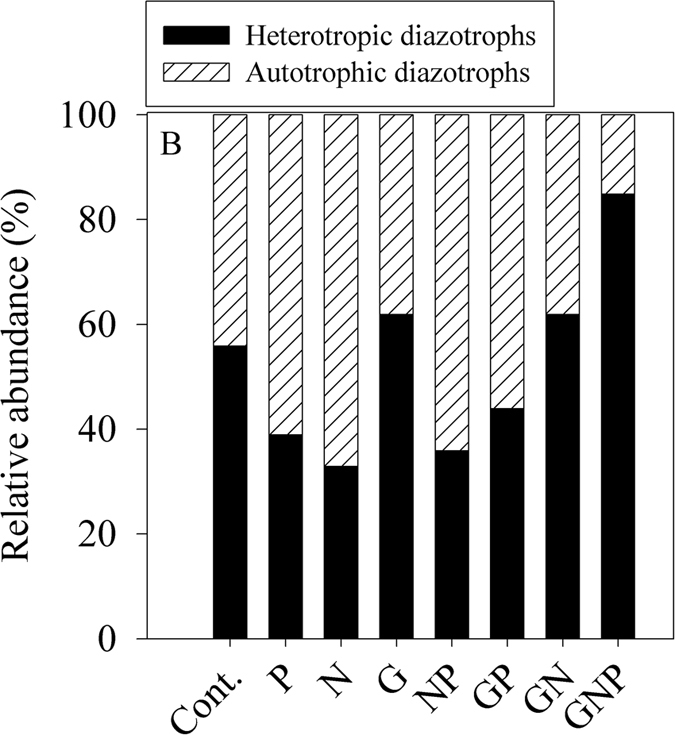
The relative abundance of phototrophic and heterotrophic *nifH* cDNA OTUs. Samples were collected from microcosms that underwent 48 h of incubations in complete dark + DCMU conditions under various nutrient-enriched scenarios. The phototrophy—heterotrophy nature of the *nifH* OTUs was determined by clustering to the known sequences listed in Table S1, similar to the analysis performed in Fig. 1.

Furthermore, based on *nifH* gene expression, 56% of the active diazotrophic communities in the control bottle microcosms were heterotrophs following two days of dark conditions (Fig. 3). These clusters were represented mostly by alpha-Proteobacteria phylotypes as well as by marine Stromatolites; a complex community of benthic heterotrophic Protobacteria and phototrophic heterocyst-forming cyanobacteria^53^. In the glucose supplemented microcosms (G, GN, GP and GNP) the relative abundance of these heterotrophic *nifH* expression bacteria increased up to 85% highlighting their importance to N_2_ fixation (Fig. 3).

The biochemical and phylogenetic results indicate that during the summer period at the EMS coastline, active diazotrophs are mostly heterotrophs and often limited by organic carbon. Furthermore, our results point on a strong dependence between the availability of organic carbon in the form of glucose, bacterial metabolism and N_2_ fixation by heterotrophic diazotrophs. Once monosaccharides such as glucose are available, assimilated carbon can be directed to meet the energetic demands of the nitrogenase complex and prompt N_2_ fixation^1^. The above results are in agreement with reports from other aquatic regions such as the South Pacific Ocean^12^,^13^,^14^ and from oxygen minimum zones^15^,^16^.

Glucose metabolism by bacterial heterotrophs may cause a rapid uptake of inorganic nitrogen and phosphorus, thus leading to nutrient stress. Therefore, when glucose is available in addition to phosphorous and/or nitrogen, the activity of bacteria, including of heterotrophic diazotrophs, intensifies, resulting in high N_2_ fixation rates (Fig. 2F). This trend was also observed following addition of a mixture of amino-acids (typically composed by organic carbon and nitrogen) to surface water in the Solomon and Bismarck seas^13^, the aphotic water in the eastern Tropical south Pacific Ocean^15^ and the northern Red Sea^29^.

**Figure 4:**
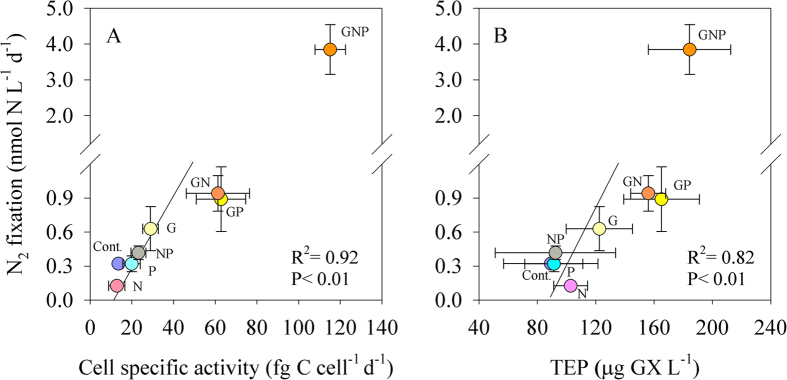
The relationship between N_2_ fixation and heterotrophic cell-specific activity (**A**) and TEP **(B**). Microcosms were conducted under dark + DCMU incubations for 48 h. Each nutrient addition type has a different color-code. Note that the GNP treatments were not included in the linear correlation. A Pearson linear correlation was carried out between N_2_ fixation and bacterial cell specific activity and TEP (P < 0.05).

The dependence on organic carbon and nutrient availability was further highlighted by the positive linear correlation between heterotrophic cell-specific activity (bacterial production per cell) and N_2_ fixation (Fig. 4A). This relationship points that supplementing the EMS water with glucose enhanced heterotrophic bacterial cell specific activity, which was tunneled into the energetically expensive N_2_ fixation process^1^. Taken together the above results indicate on a complex regulation of heterotrophic or possibly mixotrophic diazotrophs activity by varying nutrients and combinations, but primarily due to organic carbon availability.

Another indication of the importance of organic carbon to diazotrophy is the positive and significant relationship between N_2_ fixation and carbon-rich polysaccharides in the form of TEP measured in the different microcosms (Fig. 4B). TEP are acidic polysaccharide hydrogels, intensely sticky, three-dimensional-supramolecular networks, ranging in size from ~0.4 to >200 μm, and ubiquitous in marine^54^ and freshwater^38^ environments. TEP may serve as a surface and substrate for planktonic organisms such as heterotrophic bacteria and cyanobacteria, thus forming microenvironments of intense microbial activity with high carbon to nitrogen content^40^,^54^. A recent study has suggested that these carbon-rich hydrogels may also act as favorable “hotspots” for diazotrophy in oligotrophic conditions^8^.

### Impact of carbon-rich polysaccharides on heterotrophic N_2_ fixation

**Figure 5:**
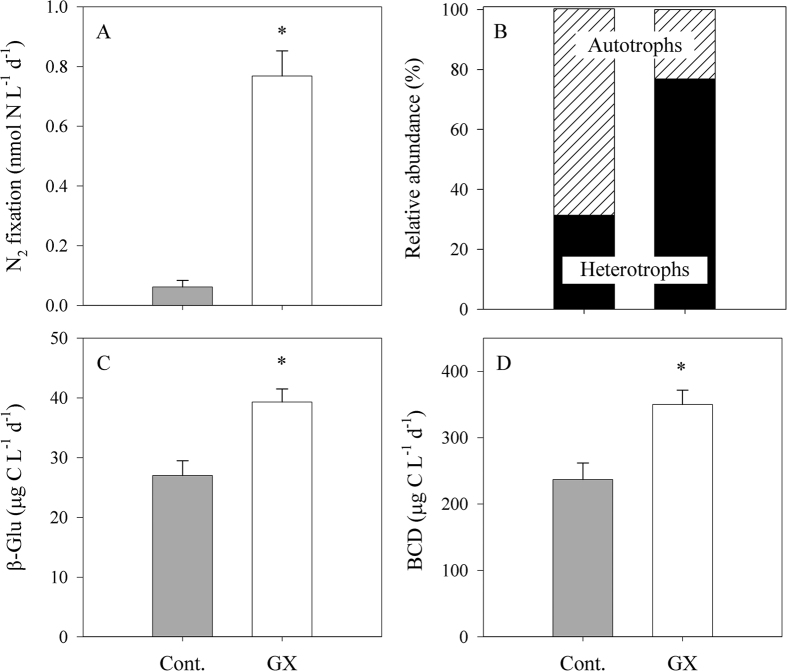
Physiological response of GX additions (TEP proxy) on N_2_ fixation. Changes in N_2_ fixation (**A**), the proportion of the *nifH* expressing phototrophic and heterotrophic diazotrophs detected in the recovered cDNA (**B**), β-glucosidase activity rates (**C**) and bacterial carbon demand (**D**) following GX addition. Bottles were incubated for 48 h under dark + DCMU conditions. The asterisks above the columns represent significant differences (one-way ANOVA and a Fisher LSD means comparison test, P < 0.05) for mean values between additions.

Carbohydrate-microgels such as TEP were found to promote heterotrophic bacterial activity and N_2_ fixation in dedicated polysaccharide-enriched microcosms (Fig. 5). EMS water was first pre-filtered (5 μm) before they were distributed into the different microcosms, thus removing 60% of the phytoplankton biomass and reducing TEP by approximately 80% (Table 2). However, the pre-filtration step did not change the bacterial abundance (Table 2). The filtrate was then supplemented with gum xanthan (GX); a commercially available polysaccharide comprised mostly of mannose, glucuronic acid and glucose^54^. The added GX was ~50% of the TEP concentration usually found in the EMS surface^17^, thus represents a moderate yet realistic scenario.

The addition of GX as a pure TEP surrogate triggered a 10-fold increase in N_2_ fixation relative to the control microcosms following two days of dark incubations (Fig. 5A). Concurrent with the elevated N_2_ fixation, a substantial increase in the abundance of heterotrophs that expressed the *nifH* genes was found following GX addition (2.5 fold compare to the control, Fig. 5B). Similarly to the previous experiment (Fig. 3), the majority of these *nifH* expressing diazotrophs were associated with Proteobacterial phylotypes.

We suggest that the elevated N_2_ fixation rates were mediated by biodegradation and metabolism of polysaccharides such as GX by heterotrophic diazotrophs. β-glucosidase is one of various ectoenzymes that are secreted by bacteria to hydrolyze polysaccharides (such as TEP) into bio-available molecules^54^,^55^. Indeed, β-glucosidase activity increased by 45% once GX was supplemented (Fig. 5C). At the same time, bacterial production and respiration were enhanced by 80% and 50% respectively (Table 2), corresponding to a 50% increase in heterotrophic bacterial carbon demand (BCD) when compared to the control microcosms (Fig. 5D). The BCD reflects the total carbon biomass that is required to sustain the metabolic demands of heterotrophic bacteria^56^. Our results indicate that TEP hydrolysis of GX by β-glucosidase accounted for 75 μg C L^−1^ d^−1^, which was approximately 15% of the total BCD (Table 2, Fig. 5). Yet, the direct contribution of polysaccharide hydrolysis to heterotrophic diazotrophs carbon demand is currently unclear.

Based on our results, we propose three, not mutually exclusive pathways, in which TEP can support heterotrophic diazotrophy: (i) TEP are often found as bio-aggregates that are heavily colonized by bacterial communities^38^,^57^. These tight bacteria-hydrogel associations may enhance polysaccharide hydrolysis by increasing the efficiency of ectoenzymes such as β-glucosidase^58^; thereby support the high energetic requirements of N_2_ fixation^1^. (ii) TEP comprises high C:N ratio^40^,^54^. Therefore, as carbon availability enhances heterotrophic bacterial activity, nitrogen becomes the limiting factor and therefore diazotrophy is promoted^26^. (iii) It was estimated that diazotrophs (such as *Crocosphaera watsonii*) direct approximately 60% of the energetic costs of N_2_ fixation to remove intracellular oxygen since nitrogenase is an oxygen-sensitive protein^1^. Large aggregates (>1 mm) may result in oxygen gradients that decrease from the surface to the anaerobic center^59^. The anoxic conditions within large aggregates, such as TEP, may benefit heterotrophic diazotrophs by shielding the nitrogenase complex from extracellular oxygen, thus reducing the overall energetic requirements and promoting N_2_ fixation. Similarly, *Pseudomonas stutzeri* strain BAL361 isolate from the Baltic Sea surface water formed aggregates (1–4 mm), which controlled O_2_ diffusion into the cells and facilitate N_2_ fixation under an oxygenated environment^60^. We postulate that bacterial diazotrophs associated with bioavailable hydrogels are not restricted solely to TEP, but would be found with other types of bioaggregates in the aquatic environment such as marine snow, fecal pellets and detritus matter.

## Conclusions

The oligotrophic EMS is presumably an ideal environment for diazotrophy, and yet, for over a decade, only low N_2_ fixation rates have been measured throughout with no reports of basin-wide diazotrophs blooms. The results of this study indicate that N_2_ was fixed primarily by heterotrophic bacteria. Furthermore, we highlight, for the first time, that the prime limiting factor for heterotrophic bacterial diazotrophs, in the EMS is organic carbon (Figs 2 and 5) rather than phosphorus as previously suggested. In fact, we show that only when organic carbon is provided in the form of monosaccharides (glucose) or polysaccharides (e.g. TEP), which results in high C:N ratio, bacterial metabolism and biological N_2_ fixation are intensified. Such carbon-rich inputs are often occurring along the Israeli coast as well as other coastal environments along the Mediterranean Sea from various anthropogenic sources. These coastal water masses, along with its subsequent nutrients and possibly carbon-fueled diazotrophs, routinely intrudes into the open EMS and therefore may pose an important ecological role on both the coastal and open EMS water. A better understanding of the link between heterotrophic bacterial-diazotrophs and organic carbon supplements such as carbon-rich TEP hydrogels might prove critical, as climate change predicts an expansion of oligotrophy in many marine and coastal environments. Under these circumstances, microenvironments such as TEP might benefit with diazotrophs, and thereby hold important ecological significance not only to carbon sequestration but also to the nitrogen cycle.

## Additional Information

**How to cite this article**: Rahav, E. *et al*. Contribution of mono and polysaccharides to heterotrophic N_2_ fixation at the eastern Mediterranean coastline. *Sci. Rep.* **6**, 27858; doi: 10.1038/srep27858 (2016).

## Supplementary Material

Supplementary Information

## Figures and Tables

**Table 1:** The physical, chemical and biological characteristics of the EMS water collected at the study site during the summer months. Values are the averages and their standard deviations from three experiments.

**Parameter**	**Units**	**September 2013**	**August 2014**	**June 2014**
Temperature	°C	27.9	28.6	26.6
Salinity	–	39.4	39.4	39.4
Oxygen	μM	195	205	219 ± 2
NO_2_ + NO_3_	μM	0.37	0.26	0.20
PO_4_	μM	0.01	0.01	0.02
TEP	μg GX L^−1^	95 ± 20	156 ± 28	100 ± 22
Chl.*a*	μg L^−1^	0.30 ± 0.01	0.14 ± 0.03	0.22 ± 0.03
Primary production	μg C L^−1^ d^−1^	1.95 ± 0.21	1.83 ± 0.15	1.41 ± 0.15
Bacterial abundance	Cells L^−1^ × 10^8^	3.60 ± 0.52	4.95	3.56 ± 0.05
Bacterial production	μg C L^−1^ d^−1^	0.42 ± 0.07	0.67 ± 0.15	0.77 ± 0.05
N_2_ fixation	nmol N L^−1^ d^−1^	0.15 ± 0.04	0.11 ± 0.03	0.10 ± 0.02

**Table 2:** The effect of GX addition on pre-filtered (5-μm) surface water collected in June 2014 and incubated under the dark and DCMU conditions for two days.

**Parameter**	**Units**	**Initial filtered (T0)**	**Control-no addition (T48)**	**GX addition (T48)**	**GX: Control (ratio)**
Bacterial abundance	Cell × 10^8^ L^−1^	3.14	3.78 ± 0.57	6.58 ± 0.42	1.8 ± 0.3
Bacterial respiration	μg C L^−1^ d^−1^	N.A.	211 ± 26	304 ± 19	1.5 ± 0.2
Bacterial production	μg C L^−1^ d^−1^	0.48 ± 0.08	1.09 ± 0.06	1.94 ± 0.10	1.8 ± 0.2
TEP	μg GX L^−1^	19	45 ± 20	103 ± 24	2.9 ± 1.7
Chl.*a*	μg L^−1^	0.14	0.03 ± 0.01	0.02 ± 0.02	0.8 ± 1.0
Primary production	μg C L^−1^ d^−1^	0.89	0.08 ± 0.02	0.04 ± 0.01	0.5 ± 0.2

Values are the averages and their standard deviations from three replicates and their corresponding GX: Control ratio. N.A. means the data are not available. Significance was tested by one-way ANOVA and a Fisher LSD means comparison test, P < 0.05.
