# Supplementary material for: Contribution of mono and polysaccharides to heterotrophic N2 fixation at the eastern Mediterranean coastline
Source: Sci Rep. 2016 Jun 16;6:27858. doi: 10.1038/srep27858 (PMC4910064; doi:10.1038/srep27858)
Supplement: Supplementary Information [file srep27858-s1.doc]

**Contribution of mono and polysaccharides to heterotrophic N_2_ fixation at the eastern Mediterranean coastline**

**Supplementary information**

Rahav E., Giannetto M. J., and Bar-Zeev E.

**Materials and Methods**

**Primary production**

Photosynthetic carbon fixation rates were estimated using the ^14^C incorporation method^1^. Samples (50 mL) were added to polycarbonate bottles (Nalgene) in triplicates, containing 5 μCi of NaH^14^CO_3_ (Perkin Elmer, 56 mCi mmol^-1^) and incubated for 4h under ambient illumination or complete dark and under ambient temperature. Added radioactivity was tested by extracting 50 μl of each sample immediately upon addition and mixed with 50 μL of ethanolamine and stored for analysis. The incubations were terminated by filtering the spiked seawater onto Whatman GF/F filters at low pressure (<50 mmHg). The filters were incubated overnight in 5 mL scintillation vials containing 50 µl of 32% HCl to remove excess ^14^C-bicarbonate. After adding 3 mL of scintillation cocktail (Ultima-Gold) to each vial, radioactivity was measured using a TRI-CARB 2100 TR (Packard) liquid scintillation counter.

**Bacterial production**

Bacterial production rates were estimated using the ^3^H-leucine (Amersham, specific activity: 160 Ci mmol^-1^) incorporation method^2^. Samples (1.7 mL) were incubated with 100 nmol L^-1^ of [4,5-^3^H]-leucine in triplicate for 4h at ambient temperatures in the dark. Killed samples with trichloroacetic acid (Sigma T6399) were used as blanks. Samples were centrifuge twice with diluted trichloroacetic acid (5%) before added with 1 milliliter of high ^3^H affinity (Ultimata Gold) scintillation cocktail. Finally the samples were counted using a TRI-CARB 2100 TR (Packard) scintillation counter. A conversion factor of 1.5 kg C mol^-1^ per mole leucine incorporated was used, assuming an isotopic dilution of 2.0^3^.

**Nucleic acid (DNA and RNA) extraction and sequencing**

Diazotrophic diversity was determined by the amplification of the nitrogenase variable region, nifH gene in a nested PCR approach (Zehr and Turner, 2001) using an Illumina MiSeq platform at RTLGenomics (Lubbock, Texas, USA). nifH transcripts were initially amplified using the nifH3 5’-ATRTTRTTNGCNGCRTA-3’ forward primer that was constructed with (5’-3’) the Illumina i5 sequencing primer (TCGTCGGCAGCGTCAGATGTGTATAAGAGACAG). The nifH4 5’-TTYTAYGGNAARGGNGG-3’ reverse primer was constructed with (5’-3’) the Illumina i7 sequencing primer (GTCTCGTGGGCTCGGAGATGTGTATAAGAGACAG). Amplifications were performed with Qiagen HotStar Taq master mix (Qiagen Inc, Valencia, California) in 25 ul reactions. Reactions were performed under the following thermal program: 95ºC for 5 min, then 25 cycles of 94ºC for 30 sec, 54ºC for 40 sec, 72ºC for 1 min, followed by one cycle of 72ºC for 10 min and 4ºC hold.

Amplifications from the first step were added to a second PCR based on qualitatively determine concentrations. Primers for the second PCR were designed based on the Illumina Nextera PCR primers as follows nifH1 (ADNGCCATCATYTCNCC) [i5index]TCGTCGGCAGCGTC and nifH2 (TGYGAYCCNAARGCNGA) [i7index]GTCTCGTGGGCTCGG. The second stage amplification was run the same as the first stage except for 10 cycles. Amplification products were visualized with eGels (Life Technologies, Grand Island, New York). Products were then pooled equimolar and each pool was size selected in two rounds using Agencourt AMPure XP (BeckmanCoulter, Indianapolis, Indiana) in a 0.7 ratio for both rounds. Size selected pools were then quantified using the Quibit 2.0 fluorometer (Life Technologies) and loaded on an Illumina MiSeq (Illumina, Inc. San Diego, California) 2x300 flow cell at 10pM.

**Sequencing analysis**

Forward and reverse reads were combined with the Illumina paired-end read merger (PEAR)^5^. All subsequent processing was conducted in the Quantitative Insights Into Microbial Ecology (QIIME) pipeline^6^. Sequences were discarded if they had less than 200bp, an average quality score less than 25, more than 6 ambiguous bases, or identified as a chimera with the ChimeraSlayer algorithm^7^. Sequences that passed these quality filters were clustered into operational taxonomic units (OTUs), defined at 97% similarity, using the UCLUST algorithm^8^. Taxonomy was assigned with BLAST and a database of *nifH* sequences from Heller et al.,^9^. Representative sequences of each OTU were aligned with MUSCLE^10^. Samples were normalized to 200 sequences per sample (seq/sample) through a single rarefaction, and samples with fewer than 200 sequences were excluded from phylogenetic analysis.

**Table S1-** Phototrophic and heterotrophic species whose *nifH* sequences were used as outgroups in the gDNA and cDNA phylogenetic trees. ^a^ Reads that clustered with a phototrophic or heterotrophic outgroup were considered phototrophs or heterotrophs, respectively. ^b^ Outgroup used to root phylogenetic trees. ^c^ GenBank accession numbers from which sequences for outgroups were obtained.

| **Trophic Category ^a^** | **Species name** | **GenBank accession number ^c^** |
| --- | --- | --- |
| Phototrophs | *Anabaena sphaerica* ^b^ | DQ439648.1 |
|  | *Crocosphaera sp.* | KC013228.1 |
|  | *Cyanothece sp.* | AB557944.2 |
|  | *Synechocystis sp.* | AY221820.1 |
|  | *Trichodesmium sp.* | L00688.1 |
| Heterotrophs | *Desµlfovibrio vµlgaris* | AY040514.1 |
|  | *Chlorobium limicola* | AY221831.1 |
|  | *Sinorhizobium meliloti* | EU698000.1 |
|  | *Azotobacter vinelandi* | EF620496.1 |
|  | *Bradyrhizobium elkani* | KJ546610.1 |
|  | *Vibrio diazotrophicus* | U23650.1 |
|  | *Pseudomonas stutzeri* | AF117978.1 |
|  | *Dechloromonas sp.* | JX154852.1 |
|  | *Arcobacter nitrofigilis* | AY221825.1 |

**References**

1. Steemann-Nielsen, E. On the determination of the activity for measuring primary production. *J Cons Int Explor Mer* **18,** 117–140 (1952).

2. Simon, M., Alldredge, A. & Azam, F. Bacterial carbon dynamics on marine snow. *Marine Ecology Progress Series* **65,** 205–211 (1990).

3. Simon, M., Alldredge, A. & Azam, F. Protein-content and protein-synthesis rates of planktonic marine-bacteria. *Marine Ecology Progress Series* **51,** 201–213 (1989).

4. Zehr, J. P. & McReynolds, L. A. Use of degenerate oligonucleotides for amplification of the nifH gene from the marine cyanobacterium Trichodesmium thiebautii. *Applied and Environmental Microbiology* **55,** 2522–2526 (1989).

5. Zhang, J., Kobert, K., Flouri, T. & Stamatakis, A. PEAR: a fast and accurate Illumina Paired-End reAd mergeR. *Bioinformatics* **30,** 614–620 (2014).

6. Caporaso, J. G., Kuczynski, J. & Stombaugh, J. QIIME allows analysis of high-throughput community sequencing data. *Nature Methods* **7,** 335–336 (2010).

7. Haas, B. J. *et al.* Chimeric 16S rRNA sequence formation and detection in Sanger and 454-pyrosequenced PCR amplicons. *Genome Research* **21,** 494–504 (2011).

8. Edgar, R. C. Search and clustering orders of magnitude faster than BLAST. *Bioinformatics* **26,** 2460–2461 (2010).

9. Heller, P., Tripp, H. J., Turk-Kubo, K. & Zehr, J. P. ARBitrator: A software pipeline for on-demand retrieval of auto-curated nifH sequences from GenBank. *Bioinformatics (Oxford, England)* **30,** 1–8 (2014).

10. Edgar, R. C. MUSCLE: Multiple sequence alignment with high accuracy and high throughput. *Nucleic Acids Research* **32,** 1792–1797 (2004).
